# Multi-View Stereo Using Perspective-Aware Features and Metadata to Improve Cost Volume

**DOI:** 10.3390/s25072233

**Published:** 2025-04-02

**Authors:** Zongcheng Zuo, Yuanxiang Li, Yu Zhou, Fan Mo

**Affiliations:** 1 School of Design, Shanghai Jiao Tong University, Shanghai 200240, China; jason.zuo@sjtu.edu.cn; 2 School of Aeronautics and Astronautics, Shanghai Jiao Tong University, Shanghai 200240, China; 3 Xi’an Institute of Surveying and Mapping, Xi’an 710054, China; 4 Land Satellite Remote Sensing Application Center, Ministry of Natural Resources, Beijing 100048, China

**Keywords:** 3D reconstruction, drone remote sensing, multi-view stereo, feature matching, deep learning, MVSNet

## Abstract

Feature matching is pivotal when using multi-view stereo (MVS) to reconstruct dense 3D models from calibrated images. This paper proposes PAC-MVSNet, which integrates perspective-aware convolution (PAC) and metadata-enhanced cost volumes to address the challenges in reflective and texture-less regions. PAC dynamically aligns convolutional kernels with scene perspective lines, while the use of metadata (e.g., camera pose distance) enables geometric reasoning during cost aggregation. In PAC-MVSNet, we introduce feature matching with long-range tracking that utilizes both internal and external focuses to integrate extensive contextual data within individual images as well as across multiple images. To enhance the performance of the feature matching with long-range tracking, we also propose a perspective-aware convolution module that directs the convolutional kernel to capture features along the perspective lines. This enables the module to extract perspective-aware features from images, improving the feature matching. Finally, we crafted a specific 2D CNN that fuses image priors, thereby integrating keyframes and geometric metadata within the cost volume to evaluate depth planes. Our method represents the first attempt to embed the existing physical model knowledge into a network for completing MVS tasks, which achieved optimal performance using multiple benchmark datasets.

## 1. Introduction

Multi-view stereo (MVS) strives to reconstruct a dense three-dimensional model by utilizing a set of calibrated images, making it a crucial endeavor in the field of computer vision. Learning-driven MVS networks [1,2,3] have made notable advancements in terms of both the quality and efficiency of reconstruction. Commonly, these MVS networks typically employ convolutional neural networks (CNNs) to extract relevant image features and construct a cost volume through the application of the plane-sweeping algorithm [4]. Within this process, the source image undergoes warping to align with the reference view, and the subsequent regularization of the cost volume facilitates the estimation of the ultimate depth [5]. Learning-based MVS methods generally include steps such as feature extraction, cost volume construction, and cost volume regularization [2], and numerous efforts have been devoted to obtaining superior cost volume and visibility information [1]. The objective of cost volume regularization is to rectify the noise contamination within the cost volume that arises from non-Lambertian surfaces or the occlusion of objects, thereby smoothing out feature correlations.

However, learning-based MVS networks often struggle with ambiguous feature matches in reflective or texture-less regions. While cost volumes encode multi-view correspondences, they neglect geometric metadata that are critical for resolving depth ambiguities. Therefore, acquiring effective representative features and accurately matching them holds immense importance for enhancing the generalization capabilities of MVS.

Essentially, MVS tasks are one-to-many feature correspondence tasks, whereby each pixel in the reference image must be examined along the epipolar lines of all distorted source images to locate the minimal matching cost at the most suitable depth. Recent research [6,7] has underscored the significance of extensive global context in feature correspondence tasks. Nevertheless, two primary challenges arise when contemplating MVS frameworks: (a) convolution adeptly captures local features, yet the confined nature of these features hinders the comprehension of global contextual information—a pivotal aspect for robust depth assessments in demanding MVS scenarios, such as varying textures, repetitive patterns, and non-Lambertian surfaces, and (b) when determining the matching cost, features are typically extracted solely from individual images, neglecting potential inter-image correspondences. Based on the powerful feature representation ability of Transformer, our method also uses Transformer as the backbone. We liken MVS, due to its intrinsic characteristics, to a feature-matching endeavor and thus introduce a robust feature matching with long-range tracking (FMLT) approach. This method leverages both internal and external attention mechanisms to efficiently integrate contextual information from both within images and across multiple images spanning long distances.

Although camera images lack depth information, human perception systems use other scene information to infer depth, such as by comparing the distance between adjacent objects [8]. Therefore, understanding the scene structure is crucial to accurately estimate depth. To solve the difficulty of using only feature maps clues for matching, we introduce an innovative perspective-aware convolution (PAC) module that can provide more clues during the feature-matching procedure. In this study, we introduced a novel module termed PAC that was designed to augment the capacity of networks to capture features related to perspective. PAC extracts features in the depth dimension by adjusting the convolution kernel’s shape, enabling the concurrent integration of various dilation rates across convolutional branches. By embedding PAC modules within MVS networks, we empower them to produce feature maps that are sensitized to viewpoint, thereby bolstering their capability to analyze objects within scene structures specific to the view.

Recently, an array of new methods has been developed; these methods conduct reconstruction directly within the final 3D volumetric feature space [9]. Despite showcasing remarkable reconstruction outcomes, these approaches depend on computationally intensive 3D convolutional layers, constraining their utility in settings with limited resources. In this paper, we revisit fundamental principles and discover, unexpectedly, that incorporating straightforward cost-effective 2D CNNs can attain high precision in depth determination. In contrast to utilizing resource-intensive 3D convolutions, our methodology demonstrates comparable performance in 3D scene reconstruction utilizing standard truncated signed distance function (TSDF) fusion [10]. Our primary strategy includes the intelligent merging of readily available metadata into the expense volume, leading to a substantial enhancement in depth prediction and reconstruction. To evaluate our approach for depth estimation and 3D scene reconstruction, we tested it on the challenging DTU [11] and ETH3D [12] datasets and extended its application to the Tanks and Temples [13] and UrbanScene3D [14] datasets, demonstrating superior performance on randomly captured footage. Our method circumvents the computational demands of 3D convolutions, paving the way for its potential usage in embedded and resource-constrained settings.

Our most significant contributions are the following:

(1) We propose a powerful FMLT approach that leverages both internal and external attention mechanisms to integrate long-range contextual information both within individual images and across multiple images. This methodology reimagines MVS as a fundamental feature matching task and takes advantage of the remarkable feature representation capabilities offered by the Transformer architecture. By using attention mechanisms and position coding, the FMLT approach can perceive global and position-related contextual information, enhancing the robustness of depth assessments in challenging MVS scenarios.
(2) We introduce a novel module called PAC that was designed to augment the capacity to capture features related to perspective. PAC derives features along the depth dimension by modifying the shape of the convolution kernel and facilitates the parallel integration of multiple dilation rates within convolutional branches. By embedding PAC modules within MVS networks, the resulting feature maps become sensitized to the viewpoint, enhancing the analysis of objects within scene structures specific to the view. This innovation addresses the issues related to feature search and alignment, providing a more accurate and detailed reconstruction of the scene.
(3) We revisited fundamental principles and discovered that incorporating straightforward, cost-effective 2D CNNs can achieve high precision in depth determination. In contrast to resource-intensive 3D convolutions, this approach demonstrated comparable performance in 3D scene reconstruction utilizing standard TSDF fusion. The fundamental approach involves seamlessly integrating obtainable metadata within the cost volume, substantially improving the depth estimation and reconstruction quality. By eliminating the computational burden linked to 3D convolutions, this innovation is ideally suited for embedded systems and resource-limited environments.

The remainder of this paper is organized as follows: Section 2 reviews the related work in MVS and deep learning-based methods. Section 3 introduces the proposed multi-stage method in detail. Section 4 presents the experimental results and analysis. Section 5 discusses the ablation experiments used to evaluate the effectiveness of different components of the proposed method, along with a comparison of the spatial and temporal complexity of the various approaches. Finally, Section 6 concludes the paper and discusses future work.

## 2. Related Work

### 2.1. Learning-Based MVS Method

MVSNet leverages differentiable homography to encode camera parameters, thereby constructing a 3D cost volume and segmenting the MVS task into per-view depth map estimation [9]. Nevertheless, its 3D-UNet architecture for cost-volume regularization incurs substantial memory and computational demands.

To mitigate these challenges, a plethora of networks have been developed. These can be broadly categorized into RNN-based recurrent approaches [15,16] and coarse-to-fine multi-stage strategies [17,18]. The recurrent methods iteratively regularize the 3D cost volume, relying on RNNs to facilitate feature transfer across varying depth hypotheses. Due to the fact that recurrent methods exchange time for memory space, they can manage high-resolution images but suffer from reduced inference speed. Conversely, multi-stage techniques initially generate a coarse depth map and progressively refine it by narrowing the target depth range at higher resolutions, guided by preceding predictions. This coarse-to-fine approach strikes a balance between swift inference and manageable memory usage. The performance of multi-stage methods may be significantly affected by the initial conditions. If the initial conditions do not meet the requirements, it may lead to the failure or performance degradation of the subsequent processing steps.

Despite the promising advancements in learning-based MVS methods, several intractable issues persist. These include robust estimation in scenarios characterized by non-Lambertian surfaces, low texture, or significant occlusions. Ongoing research efforts are focused on addressing these limitations to further optimize the performance and applicability of learning-based MVS techniques.

### 2.2. Transformer for Feature Matching

Transformer [19] has attracted widespread attention due to its efficiency and effectiveness in natural language processing, and has recently received increasing attention in the field of computer vision [20]. Due to Transformer’s ability to utilize attention to capture global contextual information, it has been applied in feature-matching tasks.

Regarding feature alignment, SuperGlue [21] employs both self and cross-attention mechanisms to integrate the spatial configurations and visual aspects of critical points within the sparse feature alignment procedure. This strategy has exhibited remarkable proficiency, establishing itself as the current benchmark. Conversely, LoFTR [7] incorporates the Transformer architecture in a gradual refinement approach to attain precise dense alignments. Through iterative crossings of self-attention and cross-attention layers, LoFTR acquires densely populated and consistently aligned matching precursors globally. Alternatively, STTR [22] frames the stereo depth estimation as a sequence-to-sequence alignment task. It utilizes Transformers that interchangeably leverage self- and cross-attention within and across epipolar lines to discern disparities in feature descriptors, facilitating remote associations.

To address the constraints imposed by a network’s receptive field, scholars have developed diverse methodologies aimed at broadening the image area considered by the network during prediction at any given point [23]. Conventionally, expanding a network’s receptive field often entailed constructing deeper networks or augmenting the down-sampling rates. Such approaches can result in the sacrifice of subtle details captured within the feature map.

### 2.3. Cost Volume

Various techniques for depth estimation rely on calibrated stereo image pairs to determine disparities, which are subsequently translated into depth using the on-axis distance derived from the camera specifications and positioning. Early approaches compared patches [24,25,26] and the echoing methods used in optical flow estimation [27]. This early work paved the way for GCNet [28], which expanded upon prior research in planar scanned stereo [29,30] to forge the now prevalent cost volume-based depth estimation method. The standard framework involves extracting features from an input image, performing feature matching, and condensing this into a cost volume. This is followed by outputting the final disparity via a convolutional layer.

MVS tasks are comprehensive tasks aimed at determining the depth from a designated viewpoint by integrating one or more extra viewpoints captured from different positions. Normally, both the internal camera parameters (regarded as stationary) and the external information (subject to dynamic evaluation) of the reference and source views are presumed to be known. Alternatively, they can be estimated offline using techniques like structure-from-motion [31] or determined in real-time with the aid of inertial and camera tracking technologies.

Traditional MVS methods frequently employ patch correlation in conjunction with photometric consistency to produce a depth map, followed by depth merging and refinement [32,33]. Conversely, previous machine learning-based approaches involved mapping dense image features from various perspectives into a complete 3D volume. From this fused 3D volume, predictions about voxel occupancy or surface probabilities are made [34,35].

Recent studies have incorporated additional scene details to refine the cost volume, thereby enhancing the final output. Some have suggested integrating multiple reference viewpoints, such as by utilizing pooling techniques in DeepMVS [36] or averaging feature metrics in DPSNet [37,38]. Besides embedding keyframe features and values within the cost volume, depth estimation techniques also exploit temporal data in diverse manners. This includes the utilization of LSTM to fuse information across multiple frames [39,40,41] or employing test-time error reprojection for optimization [42,43]. Notably, all these approaches solely depend on color images as input, disregarding auxiliary data like relative pose estimation and viewing direction subsequent to cost-volume processing. In our research, we expanded upon the concept of the feature-matching cost volume, evolving it into a feature-matching volume that leverages easily accessible metadata to generate high-quality depth maps.

For better learning, we believe that injecting easily accessible metadata information into the cost volume will be more effective for the generalized MVS method. Therefore, we propose a new cost volume and explore how to incorporate metadata information into it to improve its applicability to and effect on MVS tasks.

## 3. Method

### 3.1. Overall Architecture Diagram

**Figure 1:**
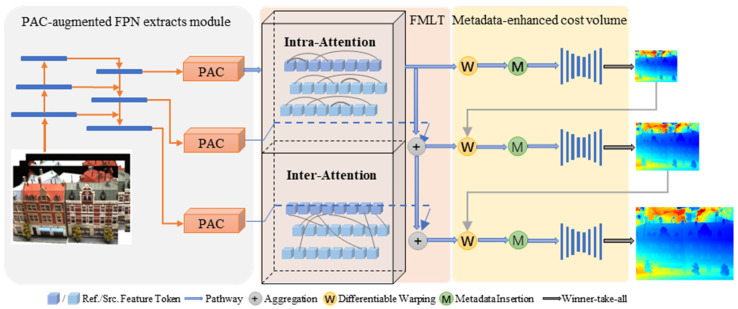
Framework of the PAC-MVSNet. The PAC-augmented FPN extracts perspective-aware features. The FMLT module integrates intra/inter-view attention. Metadata-enhanced cost volume feeds into the 2D CNN for depth regression.

In our method, given a reference image $I_{0}\in R^{H\times W\times 3}$ and its neighboring images ${\left\{I_{i}\right\}}_{i=1}^{N-1}$, as well as their corresponding camera intrinsic and extrinsic parameters, we aimed to predict a depth map aligned with $I_{0}$, and combine, refine, and merge the depth maps of all images to derive a reconstituted dense point cloud. The overall architecture is illustrated in Figure 1, which depicts the PAC-MVSNet framework. Within this framework, PAC is responsible for extracting features that are sensitive to the perspective. The FMLT module utilizes intra-attention mechanisms to integrate comprehensive contextual information within individual images. Additionally, inter-attention mechanisms are employed within the FMLT module to facilitate the efficient search and alignment of corresponding features across multiple images.

Our PAC-MVSNet initially utilizes the Feature Pyramid Network (FPN) [44] to capture multi-scale depth image characteristics across three distinct resolution tiers, spanning from rough to precise. Prior to introducing these features into Transformer, we refine the local feature capture and guarantee a seamless handoff to the Transformer using the PAC module. To harness the comprehensive contextual insights both within and between the reference and source visuals, we leveraged a FMLT module for intra-focus and inter-focus attention mechanisms. To effectively disseminate the transformed traits from lower to higher resolutions and facilitate the FMLT module’s training with gradients across all scales, we integrate all resolutions via the feature pathway.

For the N × H′ × W′ × F feature map that undergoes FMLT processing, we construct a correlation cube measuring H′ × W′ × D′ × 1 using a 3D CNN. This correlation cube serves as a foundation for subsequent regularization procedures. In this context, H′ and W′ represent the height and width of the feature map at the current stage, respectively, while F denotes the number of channels. N stands for the total number of viewpoints considered, and D′ corresponds to the relevant depth hypothesis set. Upon acquiring the regularized likelihood cube, we employ a winner-takes-all approach to pinpoint the ultimate forecast.

### 3.2. Perspective-Aware Convolution

Transformer implicitly incorporates global contextual details into feature maps through positional encoding, which can be conceptualized as a convolutional layer possessing a worldwide receptive field. Conversely, the FPN serving as the fundamental feature extractor in our proposed architecture predominantly attends to contexts within relatively confined regions. A discernible discrepancy in contextual coverage exists between these two components, posing a hindrance to both feature propagation and end-to-end learning. To overcome this drawback, we introduced a PAC module intermediary to the FPN and FMLT module. This module is capable of adaptively modulating the extent of feature extraction, bridging the gap between local and global contextual understanding. This PAC module is implemented through a regular calculation module, which can learn additional offsets of the sampling position caused by viewpoint changes and adaptively expands the receptive field according to the local environment.

Researchers have been exploring various techniques to expand a network’s receptive field, which encompasses the spatial extent of the image area that the network takes into account during the prediction process at a given point. Typically, researchers either construct deeper networks or increase the frequency of down-sampling to broaden the network’s receptive field. Nevertheless, these approaches often result in the sacrifice of delicate details within the feature maps.

**Figure 2:**
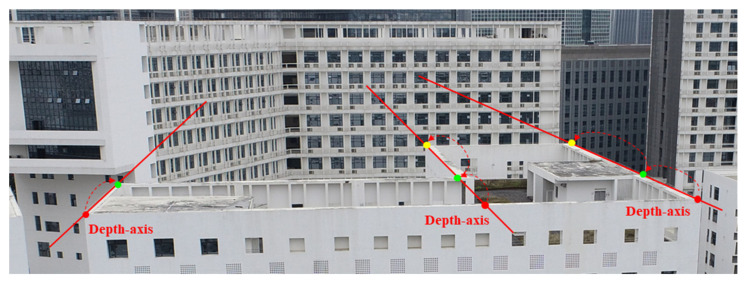
Enhancing depth prediction for a specific point by deriving the depth-axis and applying a skewed convolutional kernel.

We introduce an innovative PAC module that is specifically designed to extract features along the perspective line at each pixel position. Based on the insight that objects close to the depth axis provide crucial cues for depth estimation, Figure 2 illustrates a PAC that aids in inferring the depth of field. Directly predicting the depth of the green dot is challenging, but by determining the depth axis for each pixel and then extracting neighboring pixels both in front of and behind it along this axis, we can significantly enhance the accuracy of the depth prediction for the green dot. To achieve this, we employ the camera’s intrinsic matrix to derive the depth axis and introduce a specialized skewed convolutional kernel designed to capture pertinent features along this axis.

Standard convolutional layers often fail to capture distant dependencies within images. We strove to consciously incorporate perspective information into the network by instructing the convolution kernels to collect features along the depth axis linked to each pixel. These axes are represented by straight lines that run parallel to the camera’s depth axis within its coordinate system. Through the application of a camera pinhole model, we project these lines onto the image plane, gaining insight into how the position of each pixel will vary across the image as the depth values fluctuate. Additionally, we calculate the angle formed between the depth axis and the u-axis, referred to as the viewing angle, and leverage it to represent perspective lines.

Below, we elaborate on the process of deriving the viewing angle based on the camera pinhole model [44].

(1) $$\left[\begin{array}{c} u\\ v\\ 1 \end{array}\right]=\frac{1}{Z_{\omega }}\left[\begin{array}{ccc} f_{x} & 0 & C_{x}\\ 0 & f_{Y} & C_{Y}\\ 0 & 0 & 1 \end{array}\right]\left[\begin{array}{c} X_{\omega }\\ Y_{\omega }\\ Z_{\omega } \end{array}\right]$$

To derive the angle of view, we begin with the pinhole camera model formula. In Equation (1), $\left(X_{\omega },Y_{\omega },Z_{\omega }\right)$ denotes the point’s coordinates within the camera’s coordinate system, whereas (u,v) signifies the pixel coordinates projected onto the image plane. The parameters $f_{x}$ and $f_{Y}$ represent the focal lengths in pixels along the *u*-axis and *v*-axis, respectively. The values $C_{x}$ and $C_{Y}$ are associated with the principal point, which is the point where the optical axis intersects the image plane.

To ascertain the magnitude of pixel displacement arising from depth variations, we differentiate Equation (1) with respect to $Z_{\omega }$, yielding

(2) $$\left\{\begin{array}{c} \frac{d_{u}}{{dZ}_{\omega }}=-\frac{X_{\omega }f_{x}}{Z_{\omega }^{2}}\\ \frac{d_{v}}{{dZ}_{\omega }}=-\frac{Y_{\omega }f_{Y}}{Z_{\omega }^{2}} \end{array}\right.$$

where $\left(X_{\omega },Y_{\omega },Z_{\omega }\right)$ are the coordinates for a 3D point within the camera’s coordinate system, which is reconstructed from the pixel coordinates (u,v) through back-projection. As the depth information for each pixel remains undetermined, we make a supposition that every pixel in the image corresponds to a point on the ground plane, $Y_{\omega }$, and the fixed value of the ground plane’s height is designated as $Y_{0}$. To simplify the initial perspective calculation, we assume that each pixel corresponds to a point on a ground plane. However, this assumption is dynamically refined through cross-view attention and iterative optimization in subsequent steps, ensuring robustness even in non-planar scenarios. Utilizing Equation (2), we can reverse the projection process and map (u,v) back into the camera’s 3D coordinate frame. The formula for this back-projection is outlined below:

(3) $$\left\{\begin{array}{l} X_{0}=\frac{(u_{0}-C_{x})Y_{0}f_{Y}}{(v_{0}-C_{Y})f_{x}}\\ Y_{0}=Y_{0}\\ Z_{0}=\frac{Y_{0}f_{Y}}{v_{0}-C_{Y}} \end{array}\right.$$

where $\left(u_{0},v_{0}\right)$ denotes a specific pixel located in the image, while $\left(X_{0},Y_{0},Z_{0}\right)$ signifies the corresponding 3D point in the camera’s coordinate system obtained through back-projection. By inserting the values of $\left(X_{0},Y_{0},Z_{0}\right)$ into the placeholders $\left(X_{\omega },Y_{\omega },Z_{\omega }\right)$ in Equation (2), we can compute the necessary derivatives. The viewing angle ϕ is then determined using the subsequent formula:

(4) $$\phi =arctan2(\frac{d_{v}}{{dZ}_{\omega }},\frac{d_{u}}{{dZ}_{\omega }})$$

By employing Equations (2) and (4), we can determine the viewing angle for every pixel (u,v) within an image. This viewpoint enables us to dynamically adjust the shape of the kernel based on both the pixel coordinates and viewing perspective.

**Figure 3:**
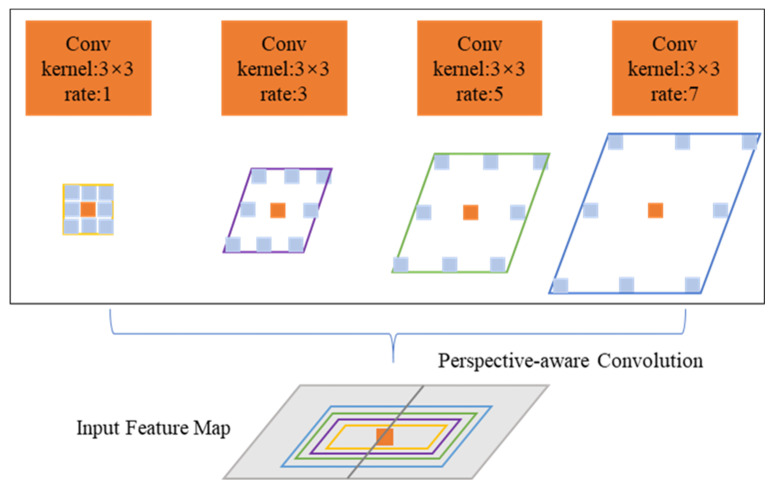
Illustration of the PAC module.

Alongside integrating a solitary PAC convolutional layer, we adopted a multi-branch architecture motivated by ASPP [45], where each branch employs distinct dilation rates. Figure 3 showcases the PAC module, which leverages parallel pathways to extract features at various scales. Nevertheless, the primary divergence stems from the kernel configuration used for feature extraction. Unlike the ASPP module that employs a standard kernel form, the PAC module incorporates an angled kernel form to direct feature extraction along the perspective line.

Our goal was to capture diverse features at varying scales along each line of perspective using the PAC module. To maintain standard functionality, we incorporated a branch armed with a conventional 3 × 3 kernel within the PAC module. This strategic decision ensures that the regular feature map remains intact as it traverses through our module. The PAC kernel dynamically skews its shape based on the viewing angle ϕ (Equation (4)). Parallel branches with dilation rates {1,3,5,7} aggregate multi-scale features along perspective lines (Figure 3). This anisotropic design bridges local FPN features and global Transformer contexts.

### 3.3. Feature Matching with Long-Range Tracking

Usually, learning-based MVS networks establish cost metrics solely from the features that have been extracted, disregarding both the global contextual data and the feature interactions among images. Nevertheless, this very information holds the key to enhancing the prediction accuracy and minimizing matching ambiguity, particularly in areas with minimal texture and repeating patterns.

**Figure 4:**
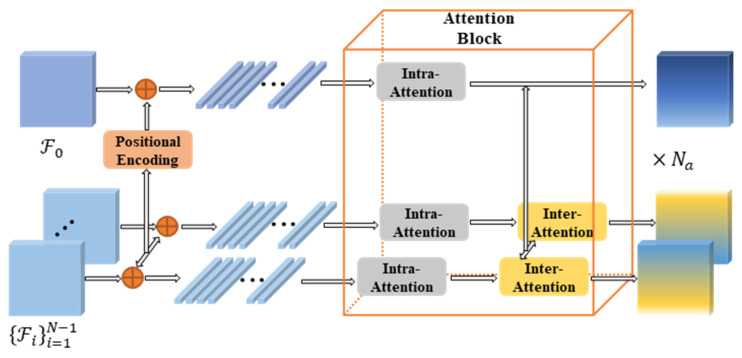
Architecture of the FMLT module. It combines intra-view self-attention for the global context and inter-view cross-attention for feature alignment. Positional encoding ensures spatial coherence across resolutions.

The Transformer-based matching method mentioned earlier handles the feature matching problem between two views. In view of the characteristics of the one-to-many matching task of MVS, we propose a FMLT approach specially customized for MVS. The structural layout of the FMLT module is illustrated in Figure 4, where positional encoding is applied to all feature maps, subsequently flattening them along these spatial dimensions. Subsequently, these attention blocks intervene and exercise intra-attention as well as inter-attention on the features.

Scaled dot-product attention mirrors the typical approach in information retrieval, whereby the characteristics are categorized into queries (*Q*), keywords (*K*), and values (*V*). The relevant data are retrieved from V by computing the dot product between *Q* and the corresponding *K* for each *V*, thereby deriving the attention weight. In a formal manner, the attention layer can be represented as

(5) $$Attention\left(Q,K,V\right)=softmax(QK^{T})V$$

The attention mechanism, among other components, assesses the similarity of features between *Q* and *K*, and retrieves the relevant information from *V* based on the determined weights. Furthermore, we employed a multi-head attention approach, dividing these feature channels into $N_{a}$ clusters (denoting the number of heads), in line with the methodology outlined in [20]. Note that the number of attention blocks $N_{a}$ was set to 6 in our implementation.

The multi-head attention mechanism [19] determines attention through the dot product of *Q* and *K*, resulting in computational expenses that scale quadratically with the input sequence length. To mitigate this computational burden, we adopted the approach outlined in [46] and employed the Linear Transformer for attention computation. The Linear Transformer modifies the conventional kernel function by utilizing

(6) $$Attention\left(Q,K,V\right)=\Phi (Q)(\Phi (K^{⊺})V)$$

where $\Phi \left(\cdot \right)=elu\left(\cdot \right)+1$ and $elu\left(\cdot \right)$ signifies the exponential linear unit activation function [47]. Given that the channel count is significantly less than the input sequence length, this modification reduces the computational complexity to a linear scale, thereby enabling the computation of attention for high-resolution images.

When the *Q* and *K* vectors originate from the same image, the attention mechanism extracts pertinent details within each perspective, effectively aggregating extensive contextual information from across the image. Conversely, if the *Q* and *K* vectors originate from different perspectives, the attention layer grasps the interconnectedness between these two viewpoints, facilitating cross-image feature integration. In the FMLT framework, we apply self-attention to both the reference image ($I_{0}$) and the set of source images (${\left\{I_{i}\right\}}_{i=1}^{N-1}$). During the calculation of cross-attention between $I_{0}$ and each $I_{i}$, the features of $I_{i}$ exclusively undergo modification.

The rationale for not updating the reference feature ($F_{0}$) based on the source feature is as follows. When aligning a reference image with its neighboring source images, maintaining the consistency of the reference features ensures a uniform target for all source features. This approach is grounded in the fundamental understanding that similarity assessments are only reliable within the confines of identical image pairs, implying that the confidence in matching varies and is not uniformly applicable across distinct image pairs.

In MVS, the conventional one-to-one correspondence task between two perspectives is superseded by the need to incorporate contextual details from multiple views to address the more complex one-to-many matching scenario. To achieve this, we introduce the FMLT approach that was designed to harness extensive contextual information both within and across images. Taking cues from [48], we incorporated positional encoding, enhancing the positional coherence and bolstering the FMLT module’s adaptability to feature maps with varying resolutions. Each view’s flattened feature map, denoted as F and belonging to the real-valued space $F\in R^{H^{\prime }W^{\prime }\times F}$, is sequentially processed by the attention blocks denoted as $N_{a}$. Inside each attention block, the initial step involves computing the self-attention between these reference features $F_{0}$ and each source feature using shared weights. This process updates all features, embedding them with their respective global contextual insights. Following this, a unidirectional cross-attention mechanism is employed, where $F_{i}$ undergoes an update based on the relevant information extracted from $F_{0}$.

### 3.4. Cost Volume Architecture

Cost volume is a 4D tensor in stereo matching, representing pixel similarities across views or frames at different disparities. Traditional stereo technology contains crucial information that frequently goes unnoticed. In our research, we integrated easily accessible metadata into the cost volumes, empowering our network to gather insights from various perspectives in a knowledgeable way. While SimpleRecon [9] pioneered metadata usage for a monocular depth, we extended this concept to multi-view stereo by integrating camera pose distances and validity masks, enabling geometric reasoning during cost aggregation. This integration can be accomplished either by explicitly appending extra feature channels or by implicitly imposing a specific feature sequence. The feature has been perspective-warped to align with the frame of the reference camera, encompassing the subsequent metadata elements.

**Figure 5:**
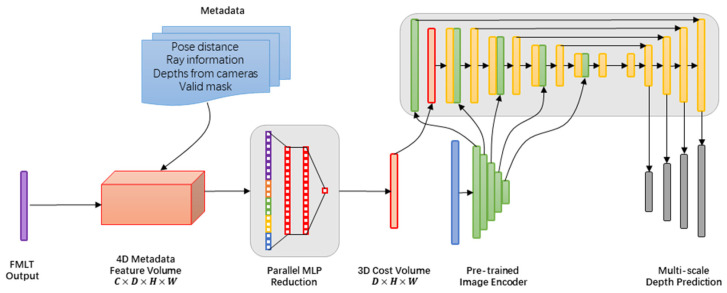
Overview of cost volume architecture.

In Figure 5, we present a depth estimation encoder–decoder architecture that integrates a cost volume, with our key innovation being the incorporation of readily available metadata into the feature volume. Each cell in this volume is then efficiently processed using an MLP to transform it into a feature map, which is subsequently input into a 2D encoder–decoder network. Our image encoder was designed to extract pertinent features from both the reference and source images, which are then utilized to populate the input cost volumes. Following this, a 2D convolutional encoder–decoder network is employed to handle the cost volume’s output, bolstered by image-level features derived from a distinct, pre-trained image encoder.

Our core innovation lies in integrating prefabricated metadata into the cost volumes alongside standard depth image features. This allows the network to tap into valuable information, including geometry and relative camera positioning data. Our model markedly surpasses prior approaches in depth forecasting, eliminating the reliance on costly 4D cost volume reduction and intricate temporal fusion.

The dot product of features is the scalar product between the characteristics of the reference image and those of the distorted source image, that is, $F^{0}\cdot {\langle F\rangle }^{n}$. Typically, this is used as an affinity metric for unique matches in the cost volume. The ray directions $r_{k,i,j}^{0}$ and $r_{k,i,j}^{n}\in R^{3}$ represent the normalized 3D position from the camera origin to the plane scan midpoint (k,i,j) direction. The depth of the reference plane, denoted as $z_{k,i,j}^{0}$, signifies the perpendicular distance from the reference camera to the specific point located at position (k,i,j) within the cost volume. Meanwhile, the depth obtained through reprojection in the reference frame, indicated as $z_{k,i,j}^{n}$, represents the vertical distance from the 3D point positioned at (k,i,j) in the cost volume to the source camera labeled as *n*.

The relative ray angle $\theta^{0,n}$ represents the angle between $r_{k,i,j}^{0}$ and $r_{k,i,j}^{n}$. The relative pose distance $p^{0,n}$, which quantifies the difference between the reference camera pose and each source frame pose, is given by

(7) $$p^{0,n}=\sqrt{\left\|t^{0,n}\right\|+\frac{2}{3}tr(I-R^{0,n})}$$

where t represents the translation vector, *tr* denotes the matrix trace operator, I is the identity matrix, and R is the rotation matrix. Furthermore, the depth validity mask $m_{k,i,j}^{n}$ is a binary mask indicating whether point (k,i,j) in the cost volume is projected in front of source camera *n*.

**Figure 6:**
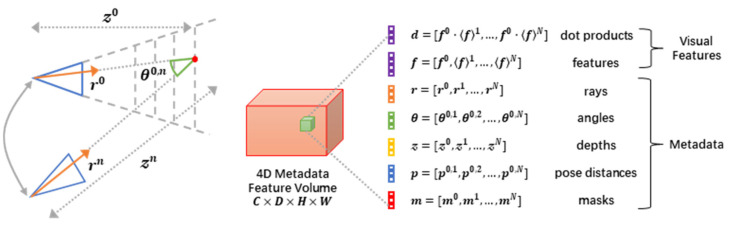
The incorporation of metadata within PAC-MVSNet.

A comprehensive summary of these traits is illustrated in Figure 6. Traditional MVS systems typically predict depth by analyzing warped features or computing disparities between features, often through techniques like dot products. In contrast, our approach augments this process by including easily obtainable metadata, aiming to enhance the overall performance. Every outcome, designated as $F_{k,i,j}$, undergoes processing via a straightforward multilayer perceptron (MLP), which yields a solitary scalar value corresponding to each coordinate (k,i,j). This scalar serves as an indication of the preliminary probability for the depth approximation of the pixel (i,j) situated on the *kth* depth plane.

We contend that by supplementing the cost volume with features derived from metadata, the MLP gains the ability to appropriately assess the significance of each source frame for every pixel location. To illustrate, think about the pose distance $p^{s,n}$; it becomes evident that for depths further away from the camera, leveraging features from the source frame with wider baselines proves more insightful. Likewise, luminance information is instrumental in discerning occlusions; discrepancies between the reference and source frame features, coupled with a substantial angle between camera rays, often indicate occlusion rather than erroneous depths. The depth validity mask aids the network in determining the reliability of source camera n’s features at (k,i,j). By granting our network access to such data, we empower it to engage in geometric reasoning during the aggregation of information from multiple sources.

Through experimental verification, the depth estimation accuracy was significantly improved by adding feature dimensions, ultimately improving the quality of 3D reconstruction. While prior research used tensors pertaining to both in-camera [49] and extra-camera [50] parameters for monocular depth estimation, we assert that our utilization of metadata constitutes a new advancement in multi-view stereo depth prediction.

### 3.5. Loss Function

Drawing inspiration from modern MVS approaches alongside monocular depth estimation strategies [51], we used a combination of geometric losses to supervise our training. We discovered that achieving the best performance necessitates a thoughtful choice of the loss function, and supervising predictions at earlier stages with smaller output scales can notably enhance the outcomes.

For depth regression loss, we employed densely supervised predictions based on log-depth. However, we utilized absolute errors for the log-depth calculations at every scale (s).

(8) $$L_{depth}=\frac{1}{HW}\sum_{s=1}^{4}\sum_{i,j}\frac{1}{s^{2}}\left|{↑}_{gt}log{\hat{D}}_{i,j}^{s}-logD_{i,j}^{gt}\right|$$

Within our methods, we employed nearest neighbor up-sampling to enhance the depth resolution from each smaller scale to the largest scale we anticipate, utilizing the ↑_*gt*_ operator. We calculate the average loss per pixel, per time interval, and per dataset batch. Through our empirical studies, we observed that this loss formulation outperforms the scale-invariant approach proposed by Eigen et al. [52], as it yields sharper depth contours, ultimately leading to superior fusion reconstruction quality.

We employed multi-scale gradient loss, along with normal loss.

(9) $$L_{grad}=\frac{1}{HW}\sum_{s=1}^{4}\sum_{i,j}\left|{\nabla ↓}_{s}{\hat{D}}_{i,j}-{\nabla ↓}_{s}D_{i,j}^{gt}\right|$$

where ∇ denotes the first-order spatial gradient ↓*s*, which signifies down-sampling in proportion to *s*. We additionally incorporated a streamlined normal loss. Herein, *N* represents the normal map, which is derived using depth and internal parameters.

(10) $$L_{normals}=\frac{1}{2HW}\sum_{i,j}\left(1-{\hat{N}}_{i,j}\cdot N_{i,j}\right)$$

For multi-view depth regression loss, we employed the ground truth depth map of every source view as supplementary guidance. This involves projecting the predicted depth, designated as the *D* plot, into each source view and subsequently calculating the average absolute error of the log depth across all valid points.

(11) $$L_{mv}=\frac{1}{NHW}\sum_{n}\sum_{i,j}\left|log{\hat{D}}_{i,j}^{0\to n}-logD_{n,i,j}^{gt}\right|$$

where ${\hat{D}}^{0\to n}$ represents the anticipated depth of the reference image, indexed at 0, which has been projected onto the source view *n*. While this notion bears resemblance to the aforementioned depth regression loss, it is solely applied to the ultimate output scale for the sake of simplicity.

The total loss is

(12) $$L=L_{depth}+\alpha_{grad}L_{grad}+\alpha_{normals}L_{normals}+\alpha_{mv}L_{mv}$$

where $\alpha_{grad}$ = 1.0, $\alpha_{normals}$ = 1.0, and $\alpha_{mv}$ = 0.2.

## 4. Experiments

### 4.1. Datasets

The DTU dataset [11] was captured under carefully controlled lighting conditions and camera positions, providing a standard dataset for the field of 3D reconstruction. It includes 128 scanned point clouds and 49 different camera viewpoints, offering a complete dataset for training the MVSNet network. To further simplify the use of this dataset, it strictly adheres to the data partitioning requirements in MVSNet: 88 scanned point clouds are used for training, and 40 for testing.

The Tanks and Temples dataset [13] was obtained under random conditions in the real world, with no restrictions on lighting conditions or camera positions, and is considered a challenging benchmark in public 3D reconstruction datasets. This dataset emphasizes the high-quality reconstruction of large scenes with complex geometries and varying textures. Therefore, it provides a sufficient testing environment for depth estimation, surface reconstruction, and texture mapping, allowing for the evaluation of our method’s performance.

The ETH3D dataset [12] contains 12 test scenes, including challenging indoor and outdoor environments. Whether it is the intricate details of indoor spaces or the vastness of outdoor landscapes, this dataset captures the characteristics of each scene with high fidelity. Additionally, the ETH3D dataset provides multi-category annotations and ground-truth depth data. Each scan includes precise depth maps and camera parameters, which facilitate accurate 3D reconstruction and analysis, and aid in evaluating the performance of different algorithms.

The UrbanScene3D dataset [14] is an emerging large-scale dataset specifically tailored for understanding urban scenes and performing 3D reconstructions. It offers a comprehensive suite of high-resolution 3D urban environments captured using advanced LiDAR scanners and RGB cameras. This dataset not only encompasses outdoor scenes but also extends to indoor spaces, providing a holistic view of urban landscapes. A key aspect of the UrbanScene3D dataset is its rich annotation, which includes semantic and instance segmentation labels, as well as 3D object annotations. These annotations facilitate the development of sophisticated 3D reconstruction algorithms that can accurately capture the geometry and semantics of urban scenes.

The DTU dataset serves as a vital benchmark for close-range multi-view 3D reconstruction; the Tanks and Temples dataset focuses on the evaluation of 3D reconstruction for outdoor objects; the ETH3D dataset is specifically tailored for indoor objects, particularly in scenarios with weakly textured surfaces; and the UrbanScene3D dataset is applicable to multi-target 3D reconstruction in large outdoor scenes. These four datasets can comprehensively assess the performance of various methods across diverse scenarios.

### 4.2. Implementation Details

To ensure the robustness and generalizability of our model, we conducted training exclusively on two datasets: the DTU training dataset and the ETH3D training dataset. These datasets provide a diverse range of scenarios and challenges, enabling us to thoroughly evaluate and fine-tune our model.

During the training and testing phases, we carefully selected the number of input images to be $N_{input}$ = 5, striking a balance between computational efficiency and informational richness. The images were processed at a resolution of 512 × 640, ensuring that sufficient detail was captured while maintaining manageable computational demands. The plane-sweeping intrinsic depth hypotheses played a crucial role in our training process, and we meticulously designed their configuration to optimize performance. Specifically, the number of hypotheses varied at each stage, with 48, 32, and 8 hypotheses at the coarsest, intermediate, and finest stages, respectively. This progressive narrowing of the hypotheses allows the model to focus on increasingly finer details as the training progresses. Correspondingly, the depth interval also progressively narrowed from the coarsest to the finest stage, with the stages decaying by factors of 0.25 and 0.5 in succession. This decaying factor ensures that the model can adaptively refine its predictions as it gains more confidence in the data. To optimize our training process, we utilized the Adam optimizer, a widely used and effective gradient-based optimization algorithm. We conducted a total of 10 training epochs; the initial learning rate was set to 0.001 and was reduced by half after the 6th and 8th epochs. This learning rate schedule ensures that the model makes steady progress during the initial stages of training while gradually refining its predictions as it approaches convergence.

The evaluation used the following metrics: (1) mean geometric distance (mm), (2) percentage of points within specific thresholds, and (3) F-score (harmonic mean of accuracy/completeness).

Our training process was conducted with a batch size of 4 across powerful NVIDIA RTX4090 GPUs. This configuration resulted in an overall training duration of approximately 8 h, with each GPU utilizing approximately 24GB of memory. Implementation was conducted using Python 3.8 with the PyTorch 2.0 deep learning framework. Regarding depth filtering and fusion, we adhered to the dynamic checking strategy outlined in [31]. This comprehensive strategy incorporates both confidence thresholds and geometric consistency checks to ensure the accuracy and stability of the results. By dynamically evaluating the confidence and consistency of our depth predictions, we can confidently identify and filter out potential outliers or inconsistencies, further enhancing the reliability and robustness of our model.

### 4.3. Experimental Results

**DTU dataset.** In our comprehensive evaluation of the PAC-MVSNet approach, we delved deeper into both the qualitative and quantitative outcomes obtained using the DTU dataset. This assessment was multifaceted, emphasizing the generation of dense and detailed point clouds, the preservation of fine-grained structures, and the overall fidelity and completeness of the 3D reconstructions.

As summarized in Table 1, PAC-MVSNet consistently outperformed the other state-of-the-art methods across a range of evaluation metrics. A closer look at the numbers reveals that PAC-MVSNet not only achieved high completeness but also maintained impressive accuracy. This is particularly noteworthy as many existing methods often struggle to balance these two critical aspects, often sacrificing one for the other. For instance, while TransMVSNet attained a respectable accuracy of 0.321, its completeness score was 0.289. Conversely, PatchMatchNet, which prioritizes completeness, had a score of 0.277 but an accuracy of 0.427. PAC-MVSNet, on the other hand, strikes a harmonious balance between these two aspects.

**Figure 7:**
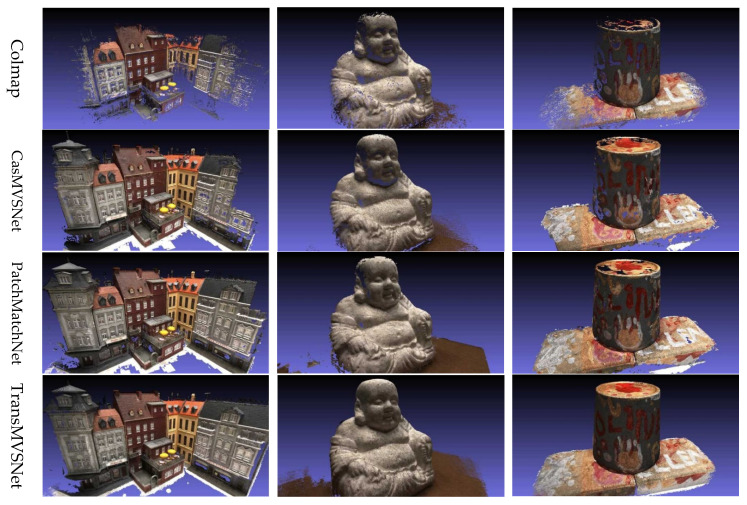

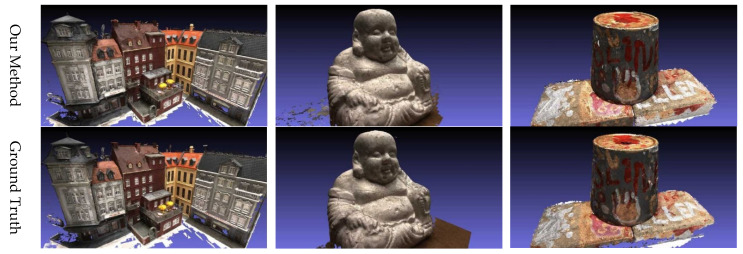
Comparison of different methods for reconstruction using the DTU dataset.

The qualitative comparison presented in Figure 7 offers a vivid visualization of PAC-MVSNet’s superiority. When pitted against Colmap [53] (traditional MVS baseline), CasMVSNet [1] (deep learning-based cascade refinement), PatchMatchNet [54] (neighborhood search optimization), and TransMVSNet [55] (Transformer-based attention mechanisms), PAC-MVSNet emerged victorious in generating high-quality point clouds, especially in complex geometries and regions with repetitive patterns. A closer examination of Figure 6 further underscores PAC-MVSNet’s strengths. The intra-attention and inter-attention mechanisms within the FMLT module were the driving forces behind the method’s exceptional performance. This holistic understanding translates into a marked improvement in the density and comprehensiveness of the generated point clouds, particularly in areas lacking texture or on intricate surfaces where the other methods faltered.

**Tanks and Temples dataset.** We evaluated the performance of PAC-MVSNet, our innovative 3D reconstruction algorithm, on the challenging Tanks and Temples dataset. This dataset is renowned for its diverse and complex scenes, making it an excellent benchmark for assessing the capabilities of various 3D reconstruction methods. Quantitatively, we relied on the F-score as our primary evaluation metric. For visual inspections, we carefully examined the reconstructed scenes for surface details, geometric accuracy, and the presence of artifacts.

As shown in Table 2, PAC-MVSNet achieved an impressive average F-score of 65.76. This score was significantly higher than that obtained by the other state-of-the-art methods, underscoring PAC-MVSNet’s superior performance across a wide range of challenging scenes. Furthermore, a breakdown of the results revealed that PAC-MVSNet consistently ranked among the top performers in all tested scenarios, demonstrating remarkable robustness and adaptability. The qualitative results further illustrate PAC-MVSNet’s superiority in outdoor scenes. This once again demonstrated the effectiveness of our perspective-aware feature-matching and cost-aggregation method utilizing metadata embedding in outdoor large scenes.

**Figure 8:**
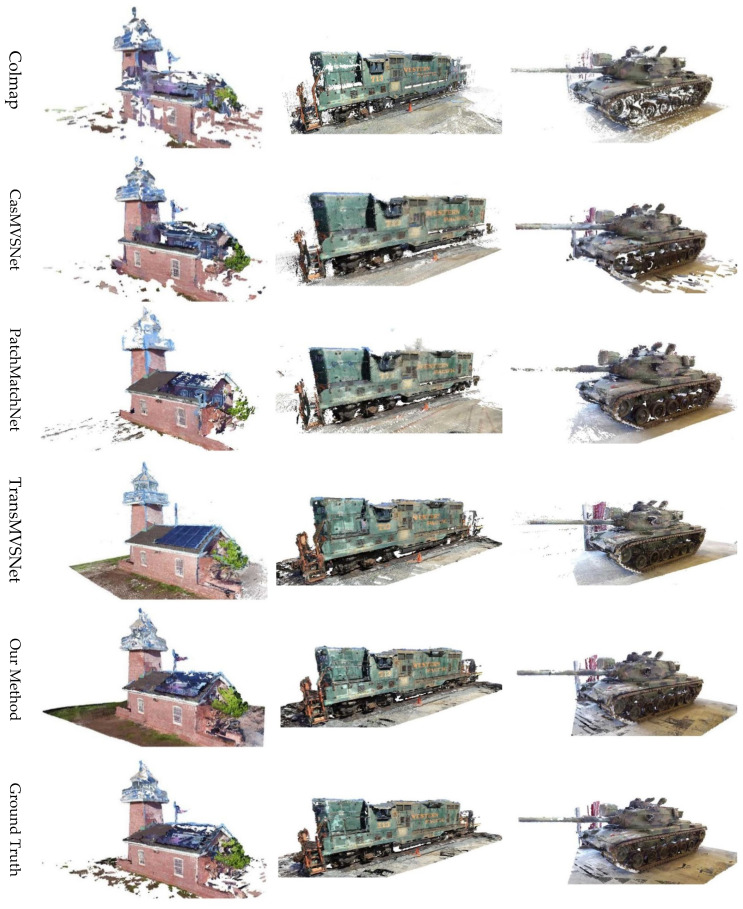
Comparison of different methods for reconstruction using the Tanks and Temples dataset.

As shown in Figure 8, the reconstructed models from PAC-MVSNet were characterized by sharp edges, smooth surfaces, and minimal voids. This is a testament to the algorithm’s ability to effectively handle complex topologies and textures, a notable challenge in 3D reconstruction.

**ETH3D dataset:** To assess the resilience of our novel approach using real-world scene datasets, we subjected PAC-MVSNet to rigorous evaluation using the high-definition ETH3D testing datasets. Comprising 13 diverse training and 12 testing scenes, the ETH3D dataset presents a formidable array of indoor and outdoor environments.

As shown in Table 3, PAC-MVSNet emerged victorious, attaining the highest F-score of 86.99 on the testing portions of ETH3D. This achievement underscores its remarkable robustness and adaptability across a wide range of scene datasets.

**Figure 9:**
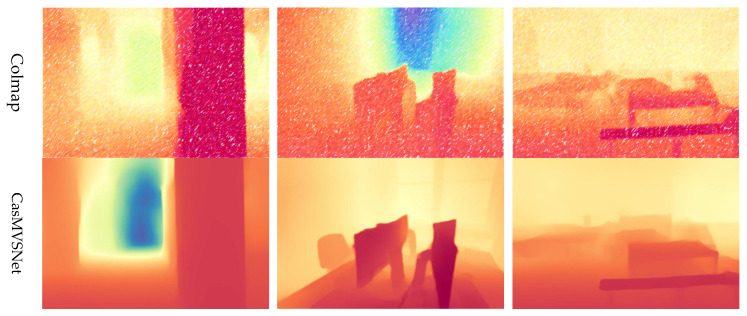

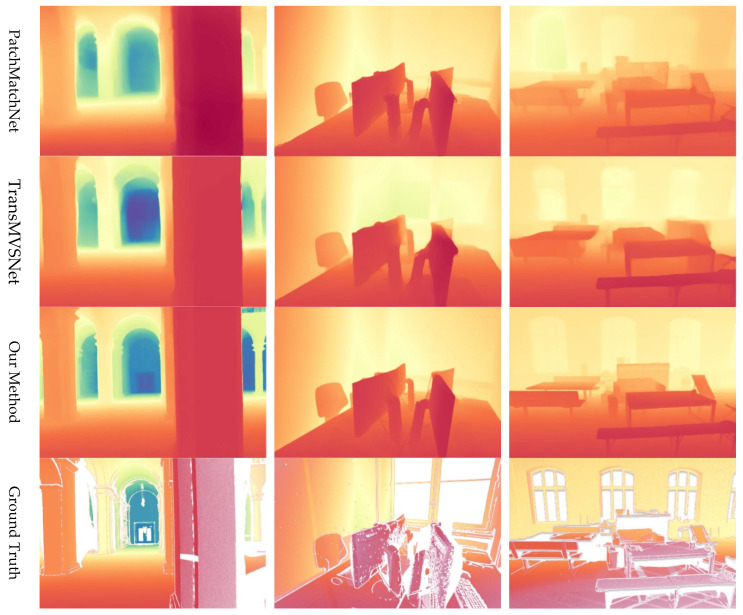
Comparison of different methods for reconstruction using the ETH3D dataset.

As shown in Figure 9, PAC-MVSNet excelled at capturing intricate details such as thin structures (e.g., chair legs) while preserving the overall layout of the scene. To further demonstrate its prowess in handling real-world obstacles such as texture-less areas and delicate structures, we pitted our reconstructed depth maps against several methods using the ETH3D benchmark. PAC-MVSNet clearly outperformed the other methods in these demanding datasets, particularly in indoor environments featuring poorly textured regions such as offices, lobbies, and chair legs. This was due to its superior feature-matching capabilities, which were enhanced by leveraging better feature-matching information. While Colmap may excel in certain well-textured datasets like the facade and court datasets, it faltered in comparison to PAC-MVSNet and TransMVSNet in more challenging datasets such as the office and electro datasets. This is due to the fact that Colmap struggles to obtain strong beliefs for inferring feature-matching selection in challenging regions. In contrast, PAC-MVSNet’s unique combination of perspective-aware matching and metadata-embedding schemes further boosts its estimation capabilities in these regions while preserving fine details.

**UrbanScene3D dataset.** In our rigorous evaluation of various 3D reconstruction techniques using the UrbanScene3D dataset, we focused on assessing the quality of the generated point clouds. This assessment encompassed both qualitative and quantitative aspects, with a particular emphasis on the density, detail, and accuracy of the reconstructions.

As shown in Table 4, our quantitative analysis revealed that among the evaluated methods, PAC-MVSNet consistently stood out in terms of point cloud quality. It achieved a superior performance across multiple evaluation metrics, including precision, recall, and overall fidelity. Notably, PAC-MVSNet excelled in preserving fine-grained structures and capturing intricate details, which is crucial for accurate urban scene reconstructions. When compared to the other state-of-the-art methods, PAC-MVSNet demonstrated a clear advantage. For instance, while Colmap and CasMVSNet produced reasonably dense point clouds, they often lacked the necessary detail and accuracy in complex urban environments. On the other hand, PatchMatchNet and TransMVSNet struggled with completeness, particularly in regions with repetitive patterns or limited texture. PAC-MVSNet, however, effectively balanced density, detail, and completeness, resulting in high-quality reconstructions.

**Figure 10:**
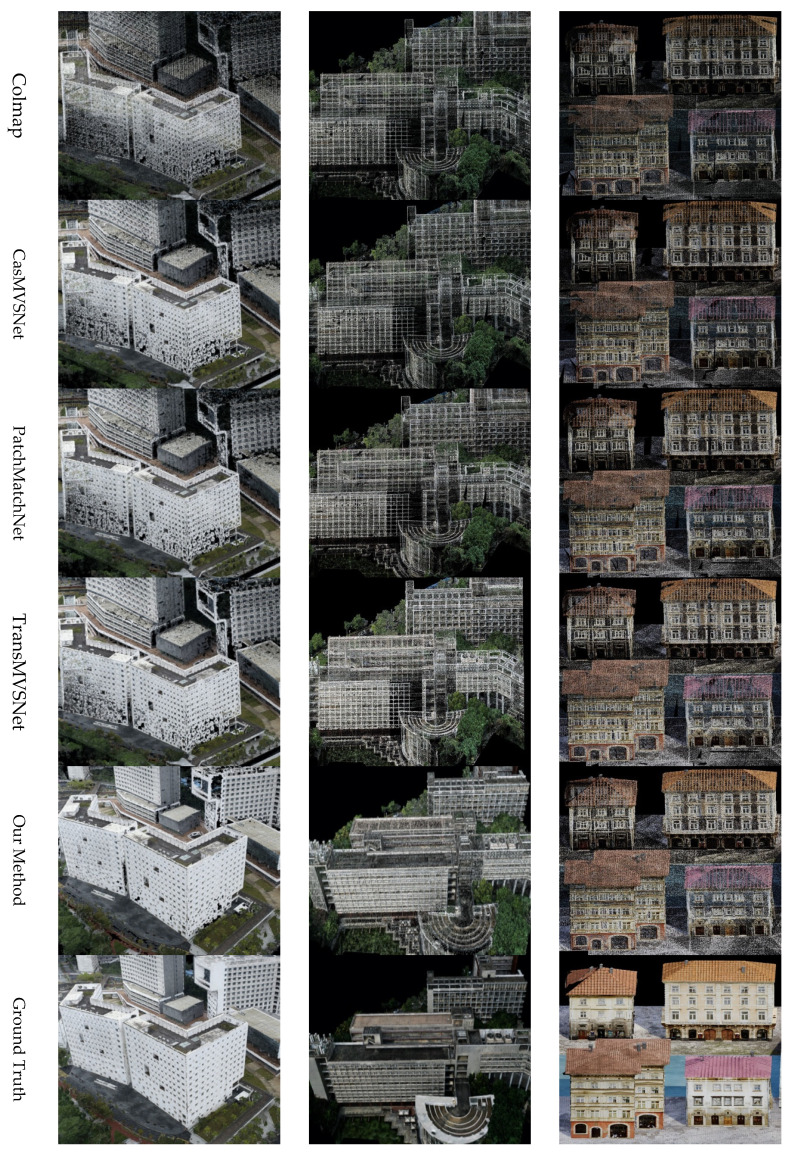
Comparison of different methods for reconstruction using the UrbanScene3D dataset.

The qualitative comparison presented in Figure 10 further illustrates PAC-MVSNet’s superiority. Its generated point clouds were not only dense but also highly detailed, accurately capturing the intricate geometries and complex structures typical of urban scenes. This is particularly evident in areas such as building facades, street furniture, and vegetation, where the other methods often faltered. The strength of PAC-MVSNet lies in its innovative approach to feature matching and cost aggregation. By incorporating metadata embedding and leveraging perspective-aware mechanisms, the method is able to effectively capture and integrate both local and global contextual information. This holistic understanding translates into superior performance in challenging urban environments, where the ability to handle complex geometries and repetitive patterns is crucial.

## 5. Discussion

### 5.1. Ablation Discussion

In this study, we evaluated the performance of three methods: our method using PAC, Metadata, and PAC + Metadata, across multiple datasets for 3D reconstruction tasks. The results consistently demonstrated that combining PAC with Metadata-based methods yielded the best performance. The results can be summarized as follows:

(1) **Complementary Effects**: Using both PAC and metadata contributed positively to 3D reconstruction, with each providing unique benefits. PAC enhances the model’s understanding of spatial relationships, resulting in improved accuracy. The use of metadata, on the other hand, offers additional context that aids in capturing finer details. When combined (Ours (PAC + Metadata)), these strategies complement each other, leading to significant improvements in reconstruction quality across all the evaluated datasets.
(2) **Performance Across Datasets:** Our method with PAC + Metadata consistently outperformed the other two methods in terms of key metrics such as mean distance, percentage of points within certain error thresholds, and F-score. This robust performance indicates the method’s strong generalization capability across diverse scenarios, including indoor scenes (DTU), outdoor landmarks (Tanks and Temples), and urban environments (ETH3D and UrbanScene3D).
(3) **Individual Contributions:** While our method with PAC generally outperformed the model with Metadata in terms of overall accuracy, the latter showed competitive results in capturing finer details, particularly in certain scenes. This suggests that PAC is crucial for improving the overall precision of the reconstruction, while the use of metadata plays a more nuanced role in enhancing surface textures and edge details. Table 1, Table 2, Table 3 and Table 4 confirmed that the use of PAC and metadata synergistically improved accuracy and completeness. PAC contributed most significantly (+12.6% F-score), validating its role in perspective alignment.

### 5.2. Inference Memory and Time Costs

We conducted a comprehensive analysis to evaluate the inference memory and time costs of our method in comparison with Colmap and other state-of-the-art MVS (multi-view stereo) techniques. Specifically, we focused on input resolutions of 1152 × 1536, as detailed in Table 5. All the experiments were conducted on a computing server equipped with an Intel Xeon 14-core E5-2680 CPU, four Nvidia RTX4090 GPU cards, and 128 GB of memory.

Our findings revealed that PAC-MVSNet excelled in terms of inference speed when compared to the other MVS methods. This advantage was primarily attributed to the efficient parallel MLP reduction incorporated into our cost volumes, which optimizes computational efficiency without sacrificing accuracy. Despite its streamlined design, PAC-MVSNet demonstrated competitive performance across various metrics, often outperforming more computationally intensive volumetric methods.

It is worth noting that the pre-trained CNN model, ResNet50, incurred significant GPU memory usage and a significant inference time. In contrast, our method and the other MVS algorithms were evaluated with a single view at a time. Among the compared methods, CasMVSNet exhibited the highest memory consumption, primarily due to the absence of group-wise pooling for the cost volume. On the other hand, while TransMVSNet boasts sophisticated features, it suffered from slow inference speeds and considerable memory demands. PatchMatchNet had a slightly better efficiency than TransMVSNet but it lagged in overall performance.

Table 5 provides a detailed breakdown of the time requirements for our method and the existing approaches across various data points. This extensive setup allowed us to thoroughly assess the performance and efficiency of each method. The results clearly indicated that our proposed PAC- MVSNet has significant advantages over the existing techniques, offering a more balanced approach between performance and computational resources.

Our method shines in terms of both run-time and memory requirements, especially at high image resolutions. For instance, at a resolution of 1536 × 1152, PAC-MVSNet reduced the memory consumption by 37.78%, 30.52%, and 6.07% compared to CasMVSNet, PatchMatchNet, and TransMVSNet, respectively. Similarly, our method achieved impressive reductions in run times, with improvements of 67.32%, 223.43%, and 6.50% over the same baselines. These findings unequivocally demonstrate the efficiency and scalability of our approach, making it a viable and attractive option for memory- and time-constrained applications.

### 5.3. Limitations

PAC-MVSNet faces challenges in highly occluded regions where conflicting depth hypotheses arise. Additionally, scenes with extreme elevation variations (e.g., aerial views) may degrade performance due to the initial ground plane assumption. Future work will integrate elevation priors and occlusion-aware attention to address these limitations.

## 6. Conclusions

We achieved three objectives: (1) robust feature matching via the PAC and FMLT approach, (2) metadata-driven cost volume enhancement, and (3) efficient 2D CNN-based depth estimation surpassing 3D counterparts. Extensive experiments validated these achievements across diverse datasets.

This article delved deeply into the core issue in MVS, which is how to establish a one-to-many feature correspondence between the reference image and all warped source images. The significance of the global context in feature-correspondence assignments was emphasized, and the challenges faced by existing MVS frameworks in processing local features and global contextual information were pointed out. To address these challenges, we proposed a Transformer-based feature-matching FMLT module that aggregates long-range contextual information both within and across images.

Furthermore, this paper highlighted the significance of understanding the scene structure in depth estimation. To overcome the difficulties of relying solely on feature maps for matching, this study innovatively proposed a PAC layer to enhance the ability of convolution to capture perspective-aware features. By modifying the shape of the convolution kernel and integrating the PAC module, this module can integrate multiple dilation rates within the convolution branch in parallel, thereby improving the MVS network’s ability to analyze objects from specific viewpoints within the scene structure.

Finally, this paper re-examined the approach of directly reconstructing in the final 3D volumetric feature space and proposed a strategy for achieving high-precision depth estimation using low-cost 2D CNNs. By cleverly integrating keyframes and geometric metadata into the cost volume, our method demonstrates a comparable performance to that of resource-intensive 3D convolution methods in 3D scene reconstruction while offering higher practicality in computationally constrained environments. The experimental results using several datasets showed that our method can effectively improve depth estimation and reconstruction quality, providing new ideas for the field of MVS. In the future, we will integrate multi-modal information like depth and optical flow to explore cross-modal perspective alignment for enhanced 3D reconstruction performance.

## Figures and Tables

**Table 1:** Quantitative results using the DTU evaluation dataset. This table contains the distance metric (where a lower score signifies better performance) and the percentage metric (in which a higher score is desirable) for all the methods, using thresholds of 1 mm and 2 mm.

Method	Mean Distance (mm)			Percentage (<1 mm)			Percentage (<2 mm)		
	Acc. ↓	Comp. ↓	Overall ↓	Acc. ↑	Comp. ↑	F-Score ↑	Acc. ↑	Comp. ↑	F-Score ↑
Colmap	0.400	0.664	0.532	71.75	64.94	68.20	84.83	67.82	75.27
CasMVSNet	0.325	0.385	0.355	69.55	61.52	65.30	78.99	67.88	72.99
PatchMatchNet	0.427	0.277	0.352	**90.49**	57.83	70.67	91.07	63.88	75.01
TransMVSNet	**0.321**	0.289	0.305	83.81	63.38	72.09	87.15	67.99	76.47
Ours (PAC)	0.342	0.262	0.302	84.45	68.37	75.52	91.72	73.16	80.78
Ours (Metadata)	0.353	0.283	0.318	83.99	66.52	74.57	89.82	72.67	79.86
Ours (PAC + Metadata)	0.331	**0.255**	**0.293**	86.46	**71.13**	**77.92**	**93.94**	**75.31**	**83.47**

**Table 2:** Comparative evaluation of reconstruction algorithms using the F-score metric on the intermediate sequences of the Tanks and Temples benchmark dataset. A higher F-score indicates superior reconstruction outcomes.

Method	Mean	Family	Francis	Horse	Lighthouse	M60	Panther	Playground	Train
Colmap	42.14	50.41	22.25	26.63	56.43	44.83	46.97	48.53	42.04
CasMVSNet	56.84	76.37	58.45	46.26	55.81	56.11	54.06	58.18	49.51
PatchMatchNet	54.03	76.50	47.74	47.07	55.12	57.28	54.28	57.43	47.54
TransMVSNet	63.52	80.92	65.83	56.94	62.54	63.06	60.00	60.20	58.67
Ours (PAC)	64.83	82.32	66.93	61.04	64.28	64.62	62.57	61.05	58.97
Ours (Metadata)	63.71	80.95	65.75	58.71	63.81	63.71	60.39	60.17	58.50
Ours (PAC + Metadata)	**65.76**	**82.43**	**67.29**	**61.72**	**65.32**	**65.71**	**62.96**	**61.28**	**59.35**

**Table 3:** An assessment of depth map generated using the high-definition multi-view test datasets from the ETH3D benchmark, displaying accuracy, completeness, and F-score (as percentages).

Method	Acc. ↑	Comp. ↑	F-Score ↑
Colmap	**91.97**	62.98	74.76
CasMVSNet	66.32	72.05	69.64
PatchMatchNet	69.71	77.46	73.38
TransMVSNet	82.23	83.75	82.98
Ours (PAC)	86.61	84.92	85.76
Ours (Metadata)	82.87	83.94	83.40
Ours (PAC + Metadata)	88.37	**85.67**	**86.99**

**Table 4:** Quantitative performance using the UrbanScene3D evaluation dataset. In this evaluation, all the methods were assessed using the distance metric, where lower values indicate superior performance, and the percentage metric, where higher values are preferred. Thresholds of 5 cm and 10 cm were applied accordingly.

Method	Mean Distance (mm)			Percentage (<50 mm)			Percentage (<100 mm)		
	Acc. ↓	Comp. ↓	Overall ↓	Acc. ↑	Comp. ↑	F-Score ↑	Acc. ↑	Comp. ↑	F-Score ↑
Colmap	79.80	89.15	84.48	68.57	54.49	60.69	81.83	62.85	71.15
CasMVSNet	58.52	84.58	71.55	65.44	57.25	61.18	76.55	65.78	70.61
PatchMatchNet	47.67	72.25	59.96	78.94	56.38	65.78	85.66	61.44	71.67
TransMVSNet	52.77	53.27	53.02	78.18	61.83	69.72	**91.23**	66.31	76.89
Ours (PAC)	45.52	42.15	43.84	81.79	71.26	76.12	90.51	74.27	81.84
Ours (Metadata)	52.03	48.76	50.40	79.63	65.34	71.58	90.23	68.21	77.56
Ours (PAC + Metadata)	**45.91**	**42.38**	**44.15**	**82.26**	**73.33**	**77.59**	90.66	**75.31**	**82.38**

**Table 5:** Representation of the model parameters and the associated memory consumption as well as time consumption when inferring 1152 × 1536 images. The results enclosed within brackets correspond to the simultaneous forwarding of 5 views, whereas those outside the brackets represent the forwarding of a single view at a time.

Method	Memory ↓ (MB)	Time ↓ (s)	Params. (Overall/Trainable) ↓ (Unit)
Colmap	4326 (9107)	0.7125 (2.9276)	---
CasMVSNet	6672 (12,351)	0.7747 (0.8311)	0.93 M/0.93 M
PatchMatchNet	6320 (11,935)	1.4975 (2.0652)	1.15 M/1.15 M
TransMVSNet	5136 (9673)	0.4931 (0.5598)	29.03 M/7.03 M
Ours (PAC + Metadata)	4842 (9251)	0.4630 (0.4972)	26.45 M/4.79 M

## Data Availability

Openly available public datasets have been utilized and are cited in the study.
